# Estimating photosynthetic capacity from leaf reflectance and Chl fluorescence by coupling radiative transfer to a model for photosynthesis

**DOI:** 10.1111/nph.15782

**Published:** 2019-04-13

**Authors:** Nastassia Vilfan, Christiaan van der Tol, Wouter Verhoef

**Affiliations:** ^1^ Wageningen University & Research Business unit Greenhouse Horticulture Droevendaalsesteeg 1 Wageningen Netherlands

**Keywords:** fluspect, leaf Chl fluorescence, photosynthesis, reflectance, Soil–Canopy Observation of Photosynthesis and Energy balance (SCOPE), *V*_cmax_

## Abstract

- In photosynthesis models following the Farquhar formulation, the maximum carboxylation rate *V*_cmax_ is the key parameter. Remote‐sensing indicators, such as reflectance *ρ* and Chl fluorescence (ChlF), have been proven as valuable estimators of photosynthetic capacity and can be used as a constraint to *V*_cmax_ estimation.
- We present a methodology to retrieve *V*_cmax_ from leaf *ρ* and ChlF by coupling a radiative transfer model, fluspect, to a model for photosynthesis. We test its performance against a unique dataset, with combined leaf spectral, gas exchange and pulse‐amplitude‐modulated measurements.
- Our results show that the method can estimate the magnitude of *V*_cmax_ estimated from the far‐red peak of ChlF and green *ρ* or transmittance *τ*, with values of root‐mean‐square error below 10 μmol CO_2_ m^−2^ s^−1^.
- At the leaf level, the method could be used for detection of plant stress and tested against more extensive datasets. With a similar scheme devised for the higher spatial scales, such models could provide a comprehensive method to estimate the actual photosynthetic capacity of vegetation.

## Introduction

Monitoring photosynthesis through remote‐sensed signals leads to a better understanding of vegetation canopies and their interaction with the environment. At least two optical indicators have been shown to respond to photosynthetic processes dynamically: Chl fluorescence (ChlF) and photochemical reflectance index (PRI). Both ChlF and PRI are intrinsically linked to photosynthesis, and have both been established as good estimators of leaf light use efficiency (LUE) and photosynthesis (for reviews, see Garbulsky *et al*., 2011; Ač *et al*., 2015). Developing methods for the estimation of photosynthesis from the two optical indicators is particularly relevant for the fast‐developing field of precision agriculture (Tremblay *et al*., 2011; Wieneke *et al*., 2016), and for the European Space Agency's dedicated Fluorescence Explorer (FLEX) satellite mission (Drusch *et al*., 2017).

In order to develop such methods, it is key to understand how the enzyme kinetics of photosynthesis are reflected in the dynamic changes of plant optical properties. The solar energy, absorbed by the leaf, undergoes one of three possible pathways: it can be used in photochemistry, emitted as ChlF, or dissipated as heat. This conversion of energy can be detected as a dynamic optical signature in the visible and near‐infrared part of the leaf spectrum.

The light is captured by the light‐harvesting antennae, and the energy is transferred to the photosynthetic reaction centres. From the captured light, *c*. 2% of light is directly emitted as ChlF. The spectrum of ChlF has a typical double peak, and ranges from *c*. 650 to 850 nm. The rest of the captured energy follows a complex process of photochemistry, eventually resulting in the fixation of CO_2_.

However, in natural conditions, plants are often exposed to various stresses that lower their capacity to utilize the available light. Excess energy must then be effectively dissipated via one of the many photoprotective mechanisms of higher plants in order to prevent damage to the photosynthetic apparatus. For example, high light exposure causes rapid saturation of the photosynthetic reaction centres, and the fastest response of the photosynthetic membrane to excess light is to dissipate the surplus of energy as heat. This process is known as the nonphotochemical ChlF quenching (NPQ). It is closely related to the xanthophyll cycle, which involves an interconversion of three xanthophylls: violaxanthin via antheraxanthin into zeaxanthin, followed by a complex series of thylakoid conformational and pH changes, concluding with heat dissipation (Demmig‐Adams & Adams, 1996; Ruban, 2016). These photoprotective mechanisms can be observed as dynamic changes in the green part of the visible spectrum, commonly expressed as the PRI (Gamon *et al*., 1992).

In order to interpret the remotely sensed data, models are needed. Simple indices using only one or two spectral bands, such as PRI, may be insufficient due to the contributions of various leaf pigments and canopy structure to the few selected spectral bands (Gitelson *et al*., 2017). Radiative transfer (RT) models can describe the light propagation within leaves and canopies based on biochemical and physical properties, enabling us to better decouple the contributions of individual parameters. Complementary to RT models, models for photosynthesis can explain the underlying biochemical processes. Coupling the two types of models would provide a unique insight into the connection between optical and biochemical properties of vegetation.

State‐of‐the‐art models, such as the Soil–Canopy Observation of Photosynthesis and Energy balance (SCOPE) model (Van der Tol *et al*., 2009), are able to quantify both the variability of photosynthesis and spectral changes at different temporal and spatial scales by employing the biochemical and RT properties of vegetation.

At the leaf level, SCOPE employs two models: a biochemical model, able to explain the relationship between ChlF, photosynthesis and NPQ under different environmental conditions (Van der Tol *et al*., 2014); and an RT model, fluspect (Vilfan *et al*., 2016). Both of the models are well tested, computationally affordable, and can function separately, providing independent outputs.

The biochemical model follows Farquhar's 1980s photosynthesis formulation (Farquhar *et al*., 1980), in which the maximum carboxylation rate *V*_cmax_ of leaves is a key parameter. *V*_cmax_ determines the maximum photosynthesis rate of a plant under optimal conditions, and it has a great influence on the modelled photosynthesis.

fluspect simulates leaf reflectance *ρ*, transmittance *τ*, and ChlF spectra as a function of leaf pigment content and structure. Recently, fluspect has been extended to simulate the dynamics of green *ρ* as a function of the xanthophyll de‐epoxidation, an RT analogy to the PRI (Vilfan *et al*., 2018).

In this study, we couple the two leaf models via ChlF and photochemical *ρ* parameters, and effectively link spectral to biochemical properties. Attempts to create such links have been made before; however, in most cases, PRI and ChlF as proxies of photosynthesis were studied separately (for a review, see Grace *et al*., 2007), with a few exceptions (Cheng *et al*., 2013; Atherton *et al*., 2016). The link of *ρ* dynamics to photochemistry is generally addressed with the use of the PRI, and relations of spectral information to photosynthetic parameters have mostly been defined via regression models (Cheng *et al*., 2013; Serbin *et al*., 2015; Zhang *et al*., 2016; Dechant *et al*., 2017; Liu *et al*., 2017). With the introduction of dynamic xanthophyll *ρ* into fluspect, we could avoid the use of PRI and approach changes in both *ρ* and ChlF in an RT‐based manner. We developed a scheme that links leaf *V*_cmax_ to *ρ*,* τ* and ChlF. Such a model has not been published before.

The coupled model enabled us to devise a method for the retrieval of the *V*_cmax_ from hyperspectral measurements of leaf ChlF and *ρ* or *τ*. In this study, we describe the coupled model and the retrieval method. We test its performance against a unique dataset, with combined leaf spectral, gas exchange and pulse‐amplitude‐modulated (PAM) measurements. We evaluate the sensitivity of the method to ChlF and *ρ*, and we discuss whether combined they provide an even better constraint to the retrieval of leaf photosynthetic parameters.

## Materials and Methods

### Laboratory experiment

The experiment was conducted in the laboratories of Forschungszentrum Jülich in February and March 2014. Sugar beet (*Beta vulgaris* L.) and barley (*Hordeum vulgare* L.) plants were grown in pots under controlled conditions in a glasshouse in Forschungszentrum Jülich between December 2013 and March 2014. Owing to the limited winter light conditions, the natural light was complemented with artificial light from growth lamps for 15 h d^−1^. The plants used in this experiment were grown under a light intensity of *c*. 1000 μmol m^−2^ s^−1^ (measured with a quantum sensor, LI‐190SL; Li‐Cor Inc., Lincoln, NE, USA). When the plants were fully grown, some of the pots grown under full light were exposed to water deficits. For a full description of growth conditions, see Vilfan *et al*. (2016). Measurements were collected on the same leaves in two separate experimental settings.

**Figure 1:**
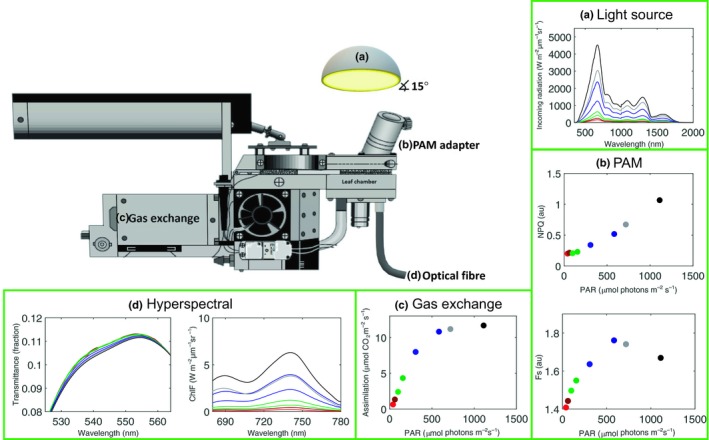
A schematic representation of the ‘chamber dataset’ measurement set‐up for a representative case of light‐response curves of a barley leaf. Samples were illuminated with a cold blue light source (a). The following measurements were taken: (b) pulse amplitude modulation (PAM) fluorometry (nonphotochemical quenching (NPQ), steady state fluorescence *F*_s_, and photochemically active radiation (PAR) are shown); (c) gas exchange (assimilation rate); and (d) spectral measurements (Chl fluorescence and transmittance). A red cut‐off filter was slotted between the light source and the sample (not shown). Gas‐exchange image used and modified by permission of Li‐Cor Biosciences.

The first set‐up, ‘Chamber dataset’, is presented in Fig. 1. It consisted of a portable gas‐exchange system (LI‐6400; Li‐Cor Inc.) connected to a (1) clear top MiniPAM Adapter (6400‐10; Li‐Cor Inc.) housing the pulse‐amplitude‐modulated fluorescence system (Mini‐PAM‐II; Heinz Walz GmbH, Effeltrich, Germany); and (2) a spectroradiometer (FieldSpec 4; Analytical Spectral Devices, Boulder, CO, USA; 350–2500 nm, 3 nm visible and near‐infrared spectral resolution). The gas‐exchange chamber bottom was equipped with an airtight slot, fitting an optical fibre of the spectroradiometer. The top could not be adjusted to house the optical fibre due to technical limitations. The chamber was illuminated externally with a cold halogen lamp (KL 2500 LCD; Schott Benelux BV, Culemborg, Netherlands). The lamp was positioned to illuminate the chamber under an angle of *c*. 15°, ensuring that the whole leaf surface within the chamber was illuminated and not shaded by the PAM optical fibre. A short‐pass filter can be slotted into the opening of the lamp, which cuts off the incoming light spectrum above *c*. 700 nm. This allowed for measurements of forward ChlF signal (‘forward’ referring to the emission being in same direction as the excitation radiation, which means in our set‐up the ChlF emanating from the abaxial side of the leaf turned away from the light source above the leaf) from *c*. 700 to 850 nm. With this set‐up, almost simultaneous measurements of passive and active ChlF, *τ*, and gas‐exchange measurements were taken under controlled conditions.

Measurements were taken on attached leaves of intact plants using one or two healthy and fully developed leaves per pot. The leaf was positioned in the chamber with its adaxial side facing the light source. The gas‐exchange system was set to a constant leaf temperature of 25°C, constant air humidity, and a CO_2_ concentration of 400 ppm. We measured both light and CO_2_ curves for each leaf. Per each leaf, the light curve was measured first, directly followed by the measurement of the CO_2_ curve, under illumination of 1000 μmol m^−2^ s^−1^. The measured leaf surface and the position of the set‐up were kept constant. Light intensities and CO_2_ concentrations were adjusted manually in the following sequences of approximately:

1. 0, 50, 100, 150, 250, 350, 500, 700, 1000, 1300 μmol m^−2^ s^−1^,
2. 400, 50, 100, 150, 250, 350, 500, 700, 900, 1200, 700, 400 ppm CO_2_.

Each measurement consisted of the following sequence of recordings: gas exchange, followed by the transmitted radiance recorded with the spectroradiometer, the filtered transmitted radiance recorded with the spectroradiometer after slotting the filter into the opening of the lamp, and the active PAM measurement at the top of the leaf after removing the filter. The procedure was repeated for each change of either illumination or CO_2_ conditions. Before each recording, we waited for the gas‐exchange conditions to stabilize, 5 min on average, and up to 20 min for a change in the illumination conditions.

A standard reflectance panel (Spectralon; Labsphere, North Sutton, NH, USA) was used separately to estimate the incident and the filtered incident radiation of the lamp and the exact transmittance of the filter for each light intensity. The panel was positioned at the same distance from the light source as the leaf.

Each radiance measurement was the average of five individual measurements over the region of 350–2500 nm, using a 136 ms integration time. Transmittance and forward ChlF spectra were calculated using the standard formulas as described in Vilfan *et al*. (2016). It should be noted that the measurements of filtered radiance below 700 nm (and consequentially the red peak of ChlF) are unreliable due to the cut‐off filter's characteristics. Moreover, the relative positioning of the reflectance panel, as well as the shape and characteristics of the gas‐exchange chamber, might have contributed to additional scattering and inaccuracies in the calculated ChlF spectra. Furthermore, many hyperspectral measurements of barley had to be excluded from the study because the leaves were too small to cover the surface of the entire leaf chamber, contaminating the measurements with additional illumination.

LUE was calculated from gas‐exchange data as:

(Eqn 1) $$\text{LUE}=\frac{A}{\text{iPAR}}$$

where *A* (μmol CO_2_ m^−2^ s^−1^) is the assimilation rate and iPAR the incoming photosynthetically active radiation (PAR; μmol m^−2^ s^−1^) (Peñuelas *et al*., 1995; Barton & North, 2001; Gitelson *et al*., 2015).

From *τ*, we calculated the PRI as (*R*_531_ − *R*_570_)*/*(*R*_531_ + *R*_570_). For a better comparison of the spectral measurements of different leaves, we normalized both the PRI and ChlF at the far‐red peak (*F*_740_) to their respective unstressed reference values (PRI′ and $F_{740}^{\prime }$). By subtracting PRI′ from all values of PRI and $F_{740}^{\prime }$) from *F*_740_, we obtained ΔPRI and Δ*F*_740_, respectively. For the light responses, PRI′ and $F_{740}^{\prime }$) were obtained from the spectrum measured at the lowest illumination (50 μmol m^−2^ s^−1^), and for CO_2_ curves from the spectrum measured at 1200 ppm CO_2_.

For PAM measurements, standard procedures were followed (instruction manual for MINI‐PAM‐II; Maxwell & Johnson, 2000). During each measurement, a short, intense pulse of light was given, from which the quantum yield of photosystem II (PS‐II) Φ_PSII_ and electron transport rate (ETR) were calculated. ETR is automatically calculated by the accompanying software of the instrument, assuming an absorption coefficient of 0.84. Φ_PSII_ reflects the proportion of light absorbed by PS‐II that is used for photochemistry and was calculated as:

(Eqn 2) $$\Phi_{\mathrm{PSII}}=\frac{F_{m}^{\prime }-F_{s}}{F_{m}^{\prime }}$$

where *F*_s_ is the steady state ChlF and $F_{m}^{\prime }$ is light‐adapted maximum ChlF yield.

In the early morning, before the start of the measurement setup, PAM measurements were taken on dark‐adapted leaves. This allowed for the calculation of minimal and maximal dark‐adapted ChlF, *F*_o_ and *F*_m_, respectively, and subsequently the NPQ:

(Eqn 3) $$\text{NPQ}=\frac{F_{m}-F_{m}^{\prime }}{F_{m}^{\prime }},$$

where *F*_m_ represents the maximal dark‐adapted ChlF yield. Because the information of PAM experiments is contained in the ratios, we normalized the signals to *F*_o_. Since ETR, Φ_PSII_ and NPQ are also outputs of the photosynthesis model, these measurements provided important additional insights into the model.

The second set‐up, the ‘FluoWat dataset’, is described in detail in Vilfan *et al*. (2016). Measurements of bidirectional leaf *ρ*,* τ*, and ChlF were collected with the FluoWat leaf‐clip, coupled to the same spectroradiometer that was used in the first set‐up. The leaf clip has two openings for the fibre optics and one light entrance, fitting both a light source at a 45° angle and a short‐pass filter (< 650 nm, TechSpec; Edmund Optics GmbH, Mainz, Germany). For more details on the FluoWat leaf clip design, see Van Wittenberghe *et al*. (2013). The samples were illuminated with the same cold light lamp as in the first set‐up. We used measurements taken under three different light intensities, with iPAR of *c*. 200, 500 and 700–800 μmol m^−2^ s^−1^.

### Leaf models in SCOPE

#### fluspect

fluspect is an RT model for the leaf, based on the model prospect (Jacquemoud & Baret, 1990), where the absorption is a function of the concentrations and specific absorption coefficients (SACs) of pigments and water. fluspect computes *ρ* and *τ* spectra from 400 to 2500 nm, as well as ChlF spectra from 640 to 850 nm. It is implemented in matlab and published under GNU General Public License at https://github.com/christiaanvandertol. Input parameters, together with their definitions and standard values, are provided in Table 1. For a published full description of the model (fluspect‐B), see Vilfan *et al*. (2016).

**Table 1:** List of parameters for the SCOPE leaf models

Parameter	Interpretation	Range	Standard value	Unit	Origin
Leaf optical: fluspect					
*C*_ab_	Chlorophyll *a *+ *b* content	0–100	40	μg cm^−2^	prospect‐D
*C*_car_	Total carotenoid content	0–30	10	μg cm^−2^	prospect‐D
*C*_ant_	Anthocyanin content	0–10	0	μg cm^−2^	prospect‐D
*C*_w_	Water content	0–0.4	0.009	cm	prospect
*C*_dm_	Dry matter content	0–0.5	0.012	g cm^−2^	prospect
*N*	Leaf mesophyll structure parameter	1–4	1.4	—	prospect
*C*_s_	Senescence material (brown pigments)	0–0.6	0	arbitrary units	prospect
*η*	Fluorescence quantum efficiency	0–0.2	0.01	—	fluspect
*C*_x_	Photochemical reflectance parameter	0–1.5	0	—	fluspect
Leaf physiology: biochemical					
*V*_cmax_	Maximum carboxylation capacity	0–250	70	μmol CO_2_ m^−2^ s^−1^	
*m*	Ball–Berry stomatal conductance param.	2–20	8	—	
Rd_param_	Parameter for dark respiration	0.001–0.03	0.015	—	
$K_{o}^{n}$	Fitting parameter for *K*_n_ (Eqn 4)	2–6	2.48	—	
*β*	Fitting parameter for *K*_n_ (Eqn 4)	0–10	0.114	—	
*γ*	Fitting parameter for *K*_n_ (Eqn 4)	0–10	2.83	—	

In this study, we use the latest version of fluspect, called fluspect‐CX (Vilfan *et al*., 2018), which is able to simulate changes in green *ρ* from *c*. 500 to 570 nm, as a function of the xanthophyll de‐epoxidation parameter *C*_x_, an RT analogy to the PRI. Moreover, in fluspect‐CX we adopted the SAC for anthocyanins as well as recalibrated SACs for chlorophylls and carotenoids from prospect‐D (Féret *et al*., 2017).

Changes in ChlF spectra can be simulated by varying *η*_I_ and *η*_II_: the fluorescence quantum efficiency parameters for PS‐I and PS‐II, respectively. In analogy to SACs, fluspect uses two spectra for the probability density function *φ* to describe the spectral distribution of emitted ChlF: *φ*_I_ and *φ*_II_, one for each of the PS‐I and PS‐II. The two functions were adopted from Franck *et al*. (2002) and can be linearly mixed. However, owing to systematic discrepancies between measured and simulated ChlF spectra, *φ* has recently been recalibrated into a single spectrum for *φ*, with a single fluorescence quantum efficiency parameter *η* (C. Van der Tol *et al*., unpublished), used in this study.

fluspect can be inverted and its parameters estimated from measured spectra. However, fluspect cannot explain the underlying processes of photosynthesis related to the dynamic parameters, such as *C*_x_ and *η*. They can, however, be estimated indirectly with the biochemical model that describes the relationship between ChlF, photosynthesis, and NPQ.

#### The biochemical model

The biochemical model used in SCOPE simulates the photosynthetic rate and fluorescence quantities as measured with PAM, as a function of absorbed light, leaf temperature, relative humidity and the concentrations of CO_2_ and oxygen (O_2_). It follows Farquhar's formulation (Farquhar *et al*., 1980), where the assimilation of CO_2_ depends on electron transport and carboxylation, and the actual rate of assimilation is determined by the most limiting of these processes. Maximum carboxylation rate per leaf area under light‐saturated conditions *V*_cmax_ is the key parameter in this model. Input parameters of the biochemical model are provided in Table 1. Only parameters relevant for this study are shown.

We used the empirical relationship between photochemical and fluorescence yield for unstressed conditions described in Van der Tol *et al*. (2014), a nonlinear relationship between the relative light saturation of photosynthesis and nonradiative energy dissipation in plants of different species calibrated to active ChlF measurements. To calculate the probability of the different fates of the excitation energy, it uses rate coefficients *K*:* K*_p_ and *K*_n_ for the photochemical ChlF quenching (PQ) and NPQ, respectively, and *K*_d_ and *K*_f_ for heat dissipation and fluorescence, respectively.

Two outputs are particularly relevant for this study: the fluorescence emission efficiency *ε*, expressed as the ratio of the steady state *F*_s_ to the dark‐adapted fluorescence yield *F*_o_; and *K*_n_, which is considered to be equivalent to NPQ, and is calculated as follows:

(Eqn 4) $$K_{n}=\frac{K_{n}^{0}\left(1+\beta \right)x^{\gamma }}{\beta +x^{\gamma }}$$

where $K_{n}^{0}$, *γ* and *β* are fitting parameters, and *x* is a measure for the light saturation of photosynthesis, calculated as:

(Eqn 5) $$x=1-\frac{\phi_{p}}{\phi_{p}^{o}}.$$

$\phi_{p}$ and $\phi_{p}^{o}$ represent the photochemical yield and the photochemical yield of dark‐adapted state, respectively.

Dark‐adapted fluorescence yield is then computed as:

(Eqn 6) $$F_{o}=\frac{K_{f}}{K_{f}+K_{p}+K_{d}}$$

and the steady state fluorescence as:

(Eqn 7) $$F_{s}=F_{m}^{\prime }\left(1-\phi_{p}\right).$$

Here, it should be noted that *F*_s_, and consequently *ε*, are related to *K*_n_ through the calculation of $F_{m}^{\prime }$:

(Eqn 8) $$F_{m}^{\prime }=\frac{K_{f}}{K_{f}+K_{d}+K_{n}}$$

### Coupling the leaf models

fluspect and the biochemical model have parameters related to photosynthesis in common, and this makes it possible to retrieve photosynthesis from the measured spectra of ChlF and *τ*. The two dynamic input parameters of fluspect,* η* (the emission efficiency of fluorescence) and *C*_x_ (the absorption SAC for the xanthophyll cycle dynamics) are related to the outputs *ε* (*F*_s_/*F*_o_) and *K*_n_ (the rate coefficient for NPQ) of the biochemical model, and the simplest possible relation is a linear one.

To couple *C*_x_ to *K*_n_, we adopt the relation of Vilfan *et al*. (2018):

(Eqn 9) $$C_{x}=0.3187\times \text{NPQ}$$

Similarly, *η* and *ε* are coupled as:

(Eqn 10) η=ε×ς

where *ς* is a scaling factor, calculated as a ratio of a typical value of *η* to a typical value of *ε*, with a value of 0.007, which represents the quantum efficiency of fluorescence in a dark‐adapted leaf. This scaling is necessary because *ɛ* = *F*_s_/*F*_o_ is a relative value, whereas *η* is an absolute emission efficiency.

### Retrieving maximum carboxylation capacity

**Figure 2:**
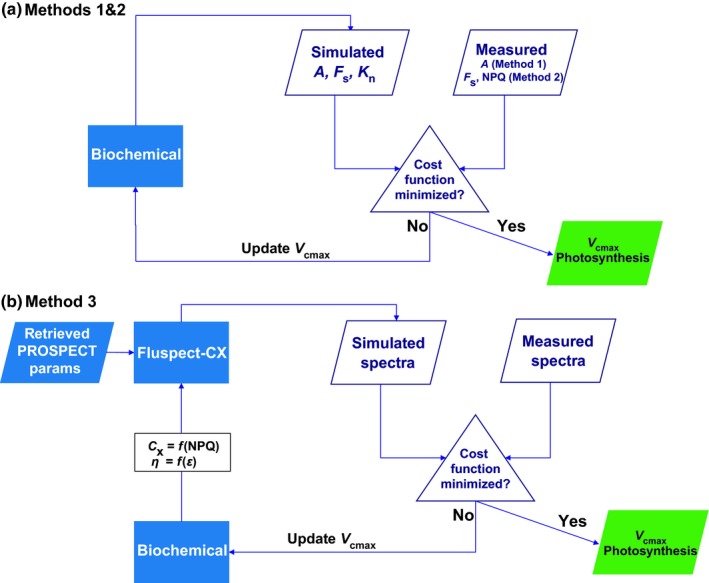
The schematics for three methods of *V*_cmax_ retrieval. (a) In methods 1 and 2, the biochemical model is inverted by constraining the inversion with either the assimilation rate *A* curves (method 1) or with nonphotochemical quenching (NPQ) and steady‐state fluorescence *F*_s_ curves (method 2). (b) In method 3, the combined model is inverted. First, the prospect parameters (see Table 1) are retrieved once per leaf. Next, the biochemical model is initialized with standard input values. Parameters for photochemical reflectance *C*_x_ and fluorescence quantum efficiency *η* are prescribed as functions of NPQ and fluorescence emission efficiency *ε*, following Eqns 9 and 10, respectively. *C*_x_ and *η*, together with the estimated prospect parameters, are provided as inputs of fluspect. The difference between the fluspect simulation and measured spectra is minimized by a cost function, resulting in the optimization of the chosen parameters.

*V*_cmax_ was retrieved in three ways, notably using gas exchange (method 1), PAM data (method 2), and hyperspectral measurements (method 3). Each method was applied to both the CO_2_ and the light response curves, resulting in six sets of values for *V*_cmax_. Method 1 (gas exchange) is the traditional way of retrieving *V*_cmax_; therefore, we used the values retrieved with this method to validate the other two methods. Fig. 2 provides an overview of these methods, Table 1 default parameters of the biochemical model, and Table 2 presents the retrieved parameters, their boundaries and constraints.

**Table 2:** List of retrieved parameters, their initial values, lower boundaries (LB), upper boundaries (UB) and constraints per each investigated method

Method	Retrieved parameter	Unit	Initial value	LB	UB	Constraint
1	*V*_cmax_	μmol CO_2_ m^−2 ^s^−1^	70	0	250	Assimilation rate curves
2	*V*_cmax_	μmol CO_2_ m^−2 ^s^−1^	70	0	250	
	$K_{n}^{0}$	—	2.48	2	6	*F*_s_ and NPQ curves
	*β*	—	0.114	0	100	
	*γ*	—	2.83	0	100	
3	*V*_cmax_	μmol CO_2_ m^−2^ s^−1^	70	0	250	ChlF and *R* or *T* spectra
	$K_{n}^{0}$	—	2.48	2	6	
	*ς*	—	0.007	0	0.2	

ChlF, Chl fluorescence; *F*_s_, steady state fluorescence; *R*, reflectance; *T*, transmittance; NPQ, nonphotochemical quenching. For a full description of parameters, see Table 1.

For method 1, we retrieved *V*_cmax_ by inverting the biochemical model only, by minimizing the squared difference between measured and simulated assimilation rates *A*, following Kosugi & Matsuo (2006), Walker *et al*. (2014), and Zheng *et al*. (2017). Measured values of PAR, leaf temperature, CO_2_ and water vapour were used to force the model.

In method 2, we minimized the quadratic difference between modelled and (PAM) measured *F*_s_ and NPQ (Fig. 2a). In order to achieve an accurate fit of measured vs modelled *F*_s_ and NPQ, we fitted not only *V*_cmax_ but also the three empirical parameters of Van der Tol *et al*. (2014) for NPQ, notably $K_{n}^{0}$, *γ* and *β* (Eqn 4). With this method we could test the potential of using steady‐state ChlF and NPQ data to retrieve photosynthesis from the biochemical model, in the absence of the informative measurements with the saturating flashes *F*_m_ and $F_{m}^{\prime }$).

In method 3, we used only the spectroscopy measurements to retrieve *V*_cmax_, which was our ultimate aim (Fig. 2b). This method enabled us to test whether *V*_cmax_ in the combined radiative transfer and biochemical model can be sufficiently constrained by passive ChlF and *τ* data. A narrow band of ChlF at the far‐red peak (730–750 nm) and of green *τ* (525–545 nm) were selected as the constraint for the model inversion. We retrieved *V*_cmax_ and the additional parameters $K_{n}^{0}$ and *ς* (Table 2) by minimizing the squared difference between the simulated and measured spectra (Eqn 11) for the whole‐light‐ or CO_2_‐response curve at once. In this retrieval, the other parameters of fluspect, notably the pigments and leaf structure parameter *N*, were kept to leaf‐specific values. These values were retrieved once per leaf before the retrieval of *V*_cmax_, $K_{n}^{0}$ and *ς*, and were assumed not to change during the light‐ and CO_2_‐response curves (Table 1). In this way, we attributed the dynamic changes to the *τ* spectrum to the xanthophyll cycle, and thus to *C*_x_ and *K*_n_ (Eqns 9, 10).

In all three methods, a trust‐region algorithm was applied in matlab using the built‐in function lsqnonlin to minimize a cost function:

(Eqn 11) $$C={\left(M-S\right)}^{2}$$

where *M* is the measured data and *S* the corresponding simulation (for the three methods: gas exchange, PAM data, and hyperspectral data). For method 3, *M* and *S* were matrices of multiple spectra: one spectrum for each of the 10 points on the CO_2_‐response curve or the eight points on the light‐response curve. These spectra included *τ* and forward ChlF. Because method 3 only uses spectra as input (and no gas‐exchange or PAM data), it was also applied to the additional FluoWat leaf clip dataset. The advantage of the FluoWat leaf clip data is that it provided not only the forward measurements (transmittance *τ* and ChlF at the shaded side), but also the backward measurements (reflectance *ρ* and ChlF at the illuminated side). However, due to some nonisotropic scattering and specular reflectance present in the *ρ* of some samples, we had to normalize the spectra of each light curve by subtracting the *ρ* at 565 nm. At this wavelength, the xanthophyll cycle ceases to have an effect on the spectra simulated by the fluspect model.

We fitted *ρ* and backward ChlF, *τ* and forward ChlF, and both *ρ* and *τ* and forward and backward ChlF together to test the dependence of the performance on the side of the leaf that is measured.

### Evaluating the model inversion

For method 1, we compared the *V*_cmax_ retrieved from the light‐response curve with those retrieved with the CO_2_‐response curves. These retrievals were then used to validate the other methods. We evaluated the goodness‐of‐fit of *V*_cmax_, *A*, and other variables by calculating the root‐mean‐squared error (RMSE) and Pearson's correlation coefficient *R* ^2^. The number of data points is slightly different among the three methods due to unrealistic values caused by occasional human error in the measurements, such as light pollution, vibrations during measurements, or incorrect measurement light and pulse settings of the PAM system.

The retrieval accuracy was evaluated for the chamber dataset with the relative RMSE. We further investigated the sensitivity of *τ* and ChlF spectra to the relevant parameters, the sensitivity of the retrievals to the starting values of the trust‐region algorithm, and the ill‐posedness of the retrieval. We calculated the Jacobian matrices *J* for *τ* and ChlF of the model for one representative sample. To obtain comparable values of the sensitivities and to normalize *J*, we multiplied *J* by the span of each parameter (see Table 2).

Error propagation in the retrieved parameters due to the measurement noise was estimated as:

(Eqn 12) $$E\left(\Delta p\Delta p^{T}\right)={\left(J^{T}J\right)}^{-1}\sigma_{r}^{2}$$

where the standard deviations of the retrieved parameters *σ*_p_ are found as the square roots of the diagonal elements of this matrix. *σ*_r_ is the SD of the measurements due to the measurement noise. For a full derivation of Eqn 12, refer to Vilfan *et al*. (2018).

## Results

### Optical and physiological measurements

**Figure 3:**
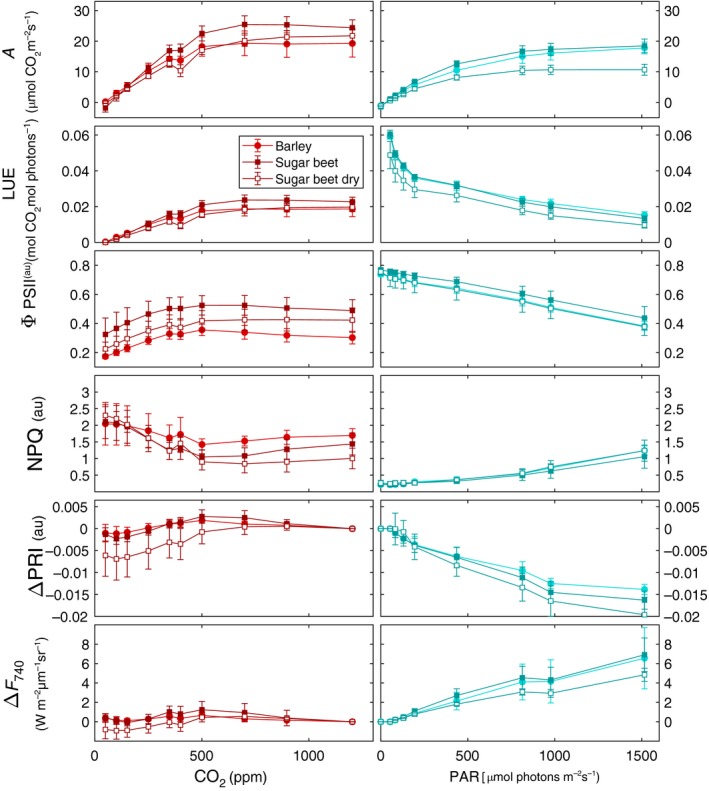
Response to changing CO_2_ concentrations and light intensity (photosynthetically active radiation, PAR) in barley (circles) and sugar beet (squares) leaves for (top to bottom): photosynthetic CO_2_ assimilation (*A*); light use efficiency (LUE) of CO_2_ assimilation; quantum efficiency of photosystem II (Φ_PSII_); nonphotochemical quenching (NPQ); photochemical reflectance index (PRI) normalized to the most nonstressed state (∆PRI); and Chl fluorescence at 740 nm normalized to the most nonstressed state (∆*F*_740_). The error bars represent ± SD from the mean. $n_{\mathrm{CO}{}_{2}}$ = 20 and *n*_light_ = 17.

In Fig. 3 we display different physiological and optical responses of leaves to variations in CO_2_ concentrations and illumination. Assimilation increases with both increasing CO_2_ and illumination intensity, until a plateau is reached. The initial slope of the assimilation curve represents the maximum LUE of the leaf, whereas the plateau signifies the light‐saturated rate of photosynthesis (Björkman, 1981).

For the case of CO_2_ curves, the indicators of photosynthetic capacity (*A*, LUE and Φ_PSII_) continuously increase until the external concentration of *c*. 700 ppm CO_2_, when the maximum assimilation and efficiency are reached. NPQ and PRI respond similarly, with the highest response at the lowest CO_2_ concentrations. The ChlF response, however, is negligible. For the case of light‐response curves, assimilation and ChlF increase with increasing light, whereas LUE, ΔPRI and Φ_PSII_ decrease_._

**Figure 4:**
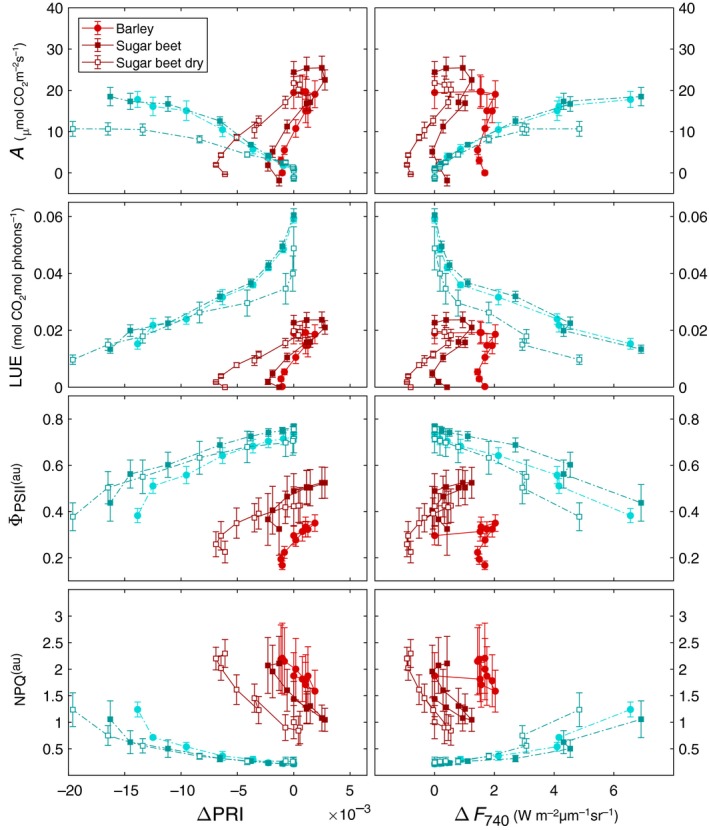
Relation of photosynthetic CO_2_ assimilation *A*; light use efficiency (LUE) of CO_2_ assimilation; quantum efficiency of photosystem II (Φ_PSII_) and nonphotochemical quenching (NPQ) to photochemical reflectance index (PRI) normalized to the most nonstressed state (∆PRI); and Chl fluorescence at 740 nm normalized to the most nonstressed state (∆*F*_740_) under changing CO_2_ concentrations (red, solid line) and light intensity (photosynthetically active radiation; blue, dotted line) in barley (circles) and sugar beet (squares) leaves. The error bars represent ± SD from the mean. $n_{\mathrm{CO}{}_{2}}$ = 20 and *n*_light_ = 17.

When comparing measured spectral responses of ΔPRI and Δ*F*_740_ to physiological variables (Fig. 4), it is immediately evident that the two types of leaf response curves do not generate the same optical response. For the light curves, dynamics of both ΔPRI and (*F*_740_ are directly driven by the increasing light intensity. Their relations with *A*, Φ_PSII_, NPQ and LUE above the peak value are almost linear, as well as their relations among each other. By contrast, the CO_2_ curves produce a seemingly less predictable response, with higher variations in measured data, and less obvious relations between the spectral and other variables.

Differences between the two species are generally small (Figs 3, 4): sugar beet (*Beta vulgaris*) seems to have better capacity for using elevated CO_2_ concentrations above 400 ppm, but light responses of the two species were similar. The difference between control and reduced soil moisture content was small; for this reason we do not differentiate between species and treatments in the following results of *V*_cmax_ retrieval.

### Retrievals of *V*_cmax_

**Figure 5:**
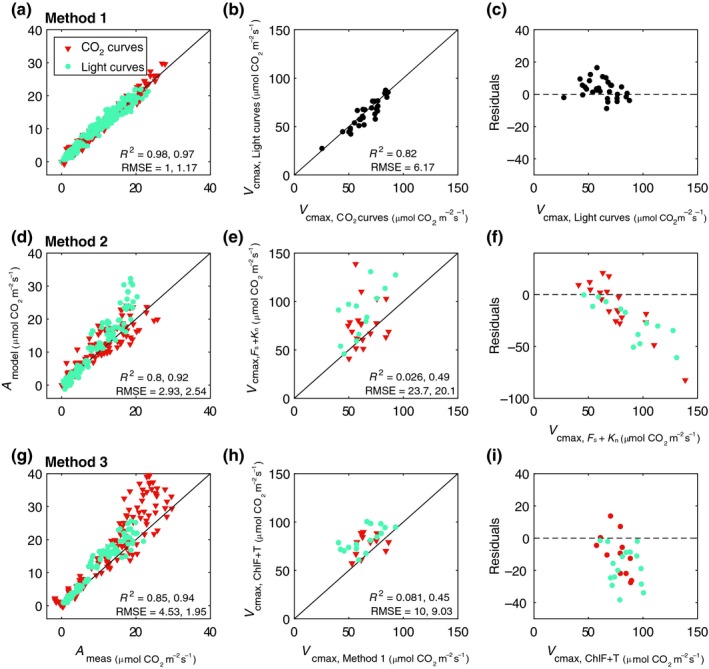
Maximum carboxylation capacity *V*_cmax_ and assimilation rate *A*, estimated with three different methods (a,b,d,e,g,h), together with residuals of predictions (measured minus estimated *V*_cmax_, c,f,i). In method 1 (a–c), we compare *V*_cmax_ retrieved from CO_2_‐response curves with the ones retrieved from light‐response curves. In methods 2 (d–f) and 3 (g–i) we compare the *V*_cmax_ retrieved from either pulse amplitude modulation measurements of steady‐state fluorescence *F*_s_ and nonphotochemical quenching *K*_n_ or hyperspectral measurements of Chl fluorescence and transmittance *τ*, respectively, with the values estimated with method 1. Number of samples (leaves) for CO_2_‐ and light‐response curves per method is *n*_m1_ = 34/34, *n*_m2_ = 19/13, and *n*_m3_ = 12/16. Pearson's correlation coefficient *R* ^2^ and RMSE are shown per measurements type, CO_2_ (red triangles) and light curves (blue circles).

*V*_cmax_ and assimilation values estimated with the three methods are presented in Fig. 5, with supporting statistical information.

In Fig. 5(b) we compare *V*_cmax_ retrieved from CO_2_‐ and light‐response curves of the same leaves with method 1. The data display a high level of correlation (*R* ^2^ = 0.82 and RMSE ≈ 6), with very accurate predictions of *A* (Fig. 5a; *R* ^2^ = 0.98 and RMSE ≈ 1).

The two other methods (Fig. 5e,h) capture the span of *V*_cmax_ for both types of response curves, with accurate predictions of *A* (Fig. 5d,g, with *R* ^2^ > 0.80 and RMSE < 4.6). Retrievals of *V*_cmax_ from PAM measurements provide the highest error (RMSE ≈ 20–23 μmol m^−2^ s^−1^). Where hyperspectral data are used to constrain the model (Fig. 5h), for both CO_2_ and light spectral responses, only the magnitude of *V*_cmax_ can be estimated (RMSE < 10), not the variation among leaves. Predictions of *A* are accurate, with *R* ^2^ > 0.85 and RMSE < 4.6.

The residuals (retrieved values minus the values retrieved from light curves with method 1) for the three methods are also shown (Fig. 5c,f,i). The smallest range of differences occurs with method 1, with unreliable estimations from method 2. Methods 2 and 3 show a consistent overestimation of *V*_cmax_, especially at high *V*_cmax_ values.

**Figure 6:**
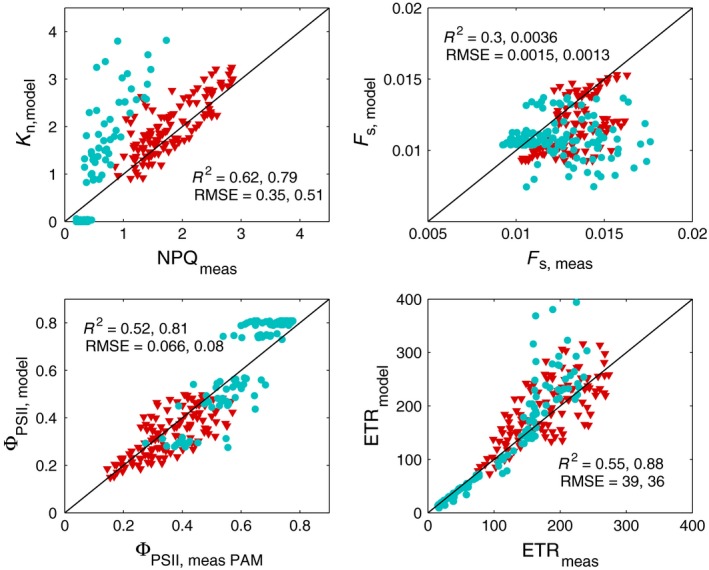
Modelled vs measured nonphotochemical quenching (*K*_*n*_ or NPQ), steady‐state fluorescence *F*_s_, quantum efficiency of photosystem II (Φ_PSII_), and electron transport rate (ETR) for method 3. Pearson's correlation coefficient *R* ^2^ and RMSE are shown per measurements type, CO_2_ (red triangles) and light curves (blue circles). $n_{\mathrm{CO}{}_{2}}$ = 12 and *n*_light_ = 16.

Retrievals from the light‐response curve have a better goodness‐of‐fit (*R* ^2^ = 0.45) than retrievals from CO_2_‐response curves (*R* ^2^ = 0.081). We evaluated other state variables besides *V*_cmax_ and *A*; Fig. 6 shows that whereas ETR, Φ_PSII_, and *K*_n_ are estimated rather well, simulated *F*_s_ is poorly correlated to PAM‐measured *F*_s_. This holds for both the CO_2_‐ and light‐response curves, although the latter have a better goodness‐of‐fit.

**Figure 7:**
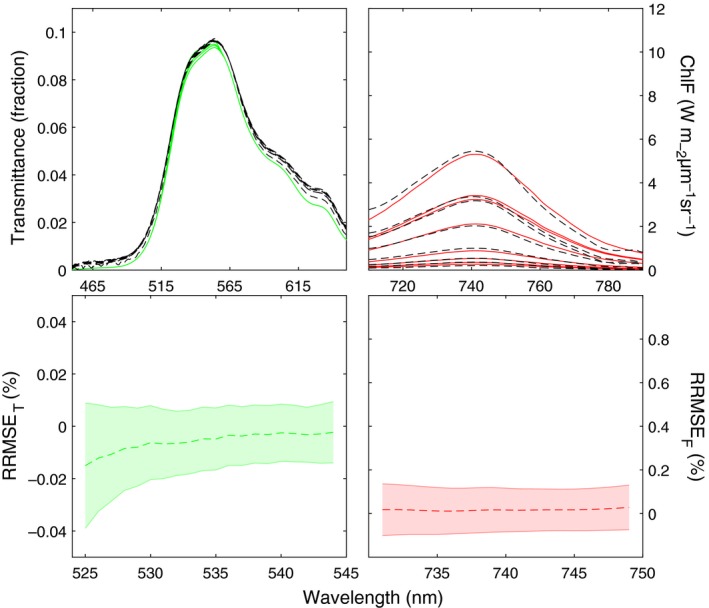
Comparison of measurements (broken line, black) and retrieval accuracy (solid line, red) after fitting with method 3 in the selected bands of transmittance (left panels) and forward Chl fluorescence (ChlF, right panels) spectra. RRMSE is the relative root‐mean‐square error of retrieval accuracy, and the shaded area represents the SD of the mean (broken line).

**Figure 8:**
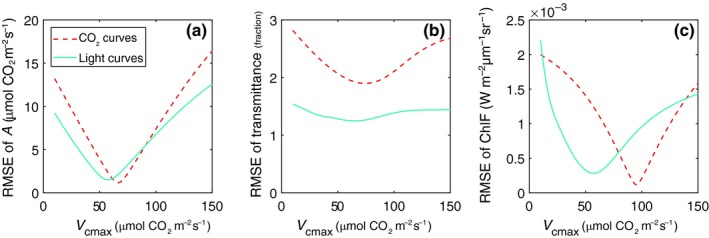
RMSE used as the cost functions for methods 1 and 3 as a function of maximum carboxylation capacity *V*_cmax_ for one representative leaf, plotted as the input range of *V*_cmax_ against the corresponding RMSE of (a) either assimilation rate (method 1) or (b) transmittance (method 3) and (c) fluorescence (method 3). The valleys of the curves represent the minima of the *V*_cmax_ optimization.

The accuracy of spectral fit after optimization between measured and simulated *τ* or ChlF is presented in Fig. 7. The spectral fit of *τ* and forward ChlF at the selected wavelengths is similarly good, with error close to 0% on average, with a maximal deviation of 4% for *τ* and 17% for ChlF. The measurement error of the spectra used for *V*_cmax_ retrieval had a negligible effect on the estimated *V*_cmax_ (3%, not shown). Most of the uncertainty in *V*_cmax_ is due to the sensitivity of *V*_cmax_ to the spectra and the model representation. This is illustrated in Fig. 8, showing the RMSE used as the cost functions for the first and third methods as a function of *V*_cmax_ for one representative leaf. Both the RMSE of fluorescence and transmittance have a single minimum, indicating their sensitivity to *V*_cmax_. The RMSE of fluorescence has a sharp and deep minimum, which confirms the sensitivity of fluorescence to *V*_cmax_, but the location of the minimum differs between the CO_2_‐ and light‐response curves, which is indicative of a model representation error. The RMSE of transmittance shows a less sharp minimum, but the location of the minimum agrees between the CO_2_ and light curves, and with the value retrieved from the assimilation data with method 1. This indicates that the model cannot fully reproduce the measured spectrum, but the transmittance nevertheless responds to *V*_cmax_ and the model is able to identify the correct value of *V*_cmax_.

**Figure 9:**
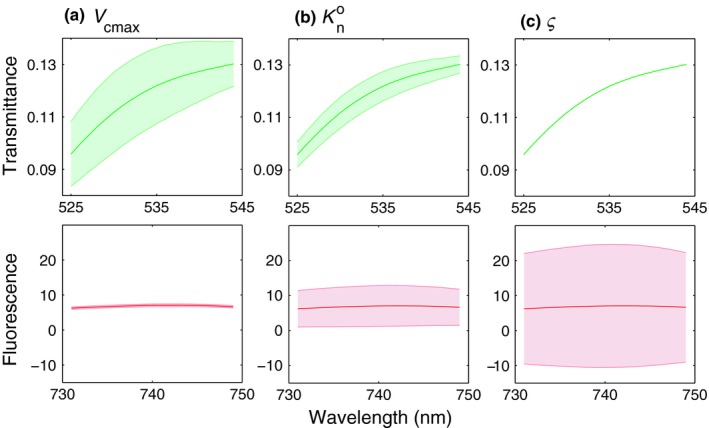
Sensitivity of transmittance (upper panels) and Chl fluorescence (ChlF, lower panels) spectra to the three fitting parameters used in method 3: (a) maximum carboxylation capacity *V*_cmax_; (b) $K_{n}^{0}$ (fitting parameter for nonphotochemical quenching *K*_n_; Eqn 4); and (c) the scaling factor *ς* that links the ChlF efficiencies of the two leaf models in Eqn 10. The shaded area denotes the span of values.

We further investigate the separate contributions of the fitting parameters used in method 3 (*V*_cmax_, $K_{n}^{0}$ and *ς*) to the spectra simulated by the combined model in Fig. 9. *τ* is influenced most by *V*_cmax_, less by $K_{n}^{0}$, and ‐obviously‐ *ς* (the scaling of ChlF) has no effect on *τ* while ChlF is influenced mostly by *ς*, less by $K_{n}^{0}$, and the effect of *V*_cmax_ is still relatively small despite the clear sensitivity shown in Fig. 8.

#### Retrieving *V*_cmax_ from FluoWat data

**Figure 10:**
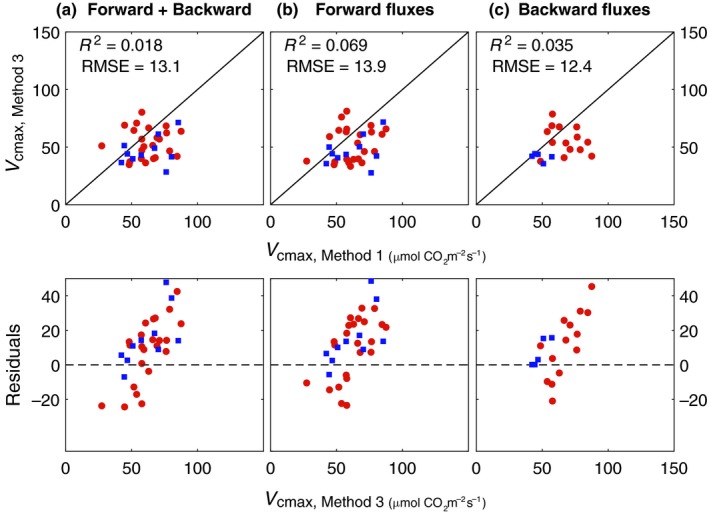
Values of maximum carboxylation capacity *V*_cmax_, retrieved for the FluoWat dataset with method 3, vs control values for barley (red circles) and sugar beet (blue squares) leaves, with the residuals (measured minus estimated *V*_cmax_) shown in the bottom panels. *V*_cmax_, $K_{n}^{0}$ (fitting parameter for *K*_n_; Eqn 4), and the scaling factor *ς* (links the Chl fluorescence (ChlF) efficiencies of the two leaf models in Eqn 10) were optimized to best reproduce measured (a) reflectance and backward ChlF, (b) transmittance and forward ChlF, or (c) all four simultaneously. Pearson's correlation coefficient (*R* ^2^) and RMSE are shown for all data combined. Note, that the control values of *V*_cmax_ (*V*_cmax_,_Method 1_) were obtained by applying method 1 to the Chamber dataset. Number of samples for barley and sugar beet per retrieval is *n*_a_ = 23/10, *n*_b_ = 23/10, and *n*_c_ = 14/5.

For the FluoWat data (Fig. 10), we obtained similar results as for the chamber dataset: the correct range of *V*_cmax_ values is retrieved (RMSE *c*. 13 μmol m^−2^ s^−1^), but with low goodness‐of‐fit. The residuals have the same span as obtained for the chamber dataset (Fig. 5h), albeit reversed: there is an increase in underestimation with increasing *V*_cmax_ values. There are no substantial differences in results when transmittance or reflectance of the leaf are used for the retrieval. It should be emphasized that we used the *V*_cmax_ values obtained earlier in the chamber for validation, because the FluoWat leaf clip does not allow for gas‐exchange measurements.

## Discussion

Both the transmittance and the fluorescence spectra (method 3) contain sufficient information to constrain *V*_cmax_, as demonstrated by the clear minima in their RMSE with respect to varying *V*_cmax_ (Fig. 8). The results show that, for our dataset, with limiting span of *V*_cmax_, the magnitude but not the variability of *V*_cmax_ among leaves can be estimated from the coupled model, by using the combined information of hyperspectral ChlF and green *ρ* or *τ*. The RMSE for the estimated *V*_cmax_ is nevertheless below 14 μmol CO_2_ m^−2^ s^−1^, which complies with the error determined by similar studies of leaf level responses (Serbin *et al*., 2012; Dechant *et al*., 2017) and by a study on airborne data by Serbin *et al*. (2015). Both Serbin *et al*. (2012) and Dechant *et al*. (2017) used a much wider range of *V*_cmax_ (0–200 μmol CO_2_ m^−2^ s^−1^). Considering the limited span of *V*_cmax_ in our measurements (50–100 μmol CO_2_ m^−2^ s^−1^), the low and comparable values of RMSE are encouraging. Leaf datasets with a known wider span of maximum photosynthetic capacity would provide a valuable further validation of the model.

The response of ChlF to changing CO_2_ is small, but nevertheless meaningful (see dashed line in Fig. 8c). This response is limited due to the fact that in a CO_2_‐response curve, a reduction of PQ is compensated by an increase in NPQ and vice versa, with limited net effect on ChlF. The responses of NPQ and ΔPRI are unambiguous, but tuning *V*_cmax_ cannot bring the RMSE of transmittance close to zero (Fig. 8b): the effect of NPQ on transmittance is small compared with the accuracy by which we can reproduce the overall transmittance spectrum.

Potential errors in the *V*_cmax_ retrieval with method 3 include measurement errors, the performance of the optimization method, and the biochemical and radiative transfer model representation. We investigated the potential effects of measurement errors and accuracy of spectral fit on the retrievals of *V*_cmax_, but these effects were minimal: up to 3% of the retrieved *V*_cmax_ values. Crucial is the model representation.

Despite controlled experimental conditions, the values of *V*_cmax_ estimated from the assimilation rates (method 1) differ up to 25% between light and CO_2_ responses (Fig. 5c). A study by Miao *et al*. (2009) has similarly shown that significant differences exist between different methods of *V*_cmax_ retrieval from *A* curves. This could be attributed to limitations of the Collatz model (Collatz *et al*., 1991) used by Van der Tol *et al*. (2014), which does not use the maximum electron transport capacity *J*_max_ of the original Farquhar model as an additional parameter. The parameterization of photorespiration may also contribute to this difference; photorespiration was not suppressed in our experiment, which was carried out under ambient O_2_.

Differences in the optimized values from transmittance and fluorescence (Fig. 8b,c) may originate from the prescribed relations of *η* and *C*_x_ to *ε* and *K*_n_, which were reconciled by using a single cost function for both transmittance and fluorescence, and allowing variations in these relationships.

We established the link between the radiative transfer model fluspect and the photosynthesis model via parameter *η* (the absolute ChlF quantum yield) to *ε* = *F*_s_/*F*_o_, and *C*_x_ to *K*_n_ via a calibrated coefficient (Vilfan *et al*., 2018). This introduces uncertainty, because PAM *F*_s_ cannot be reproduced well by the model. The poor correlation of PAM *F*_s_ to simulated *F*_s_ can at least partly be explained by the compensation of the effects of PQ and NPQ, resulting in a relatively small range of *F*_s_, which is then prone to uncertainties. This was also noted in the development of the extended biochemical model (Van der Tol *et al*., 2014, Fig. 9).

In Van der Tol *et al*. (2014), two different parametrizations are given for *K*_n_ for different datasets. Obviously, $K_{n}^{0}$, a parameter for the xanthophyll pool size, may vary. In a first attempt to fit *τ* and ChlF of the light‐response curves, we found that retrieving only *V*_cmax_ while keeping $K_{n}^{0}$ at the default value of Van der Tol *et al*. (2014) did not provide a satisfactory fit (not shown here). It was necessary, therefore, to include $K_{n}^{0}$ in the retrieval. Inclusion of *ς* was necessary in order to translate *F*_s_ in arbitrary units to absolute values of ChlF yield.

Indeed, the sensitivity analysis (Figs 8, 9) reveals that *τ* simulated with the combined model depends primarily on *V*_cmax_, whereas the magnitude of ChlF is most affected by the hitherto unconstrained *ς*, and $K_{n}^{0}$ has a similar effect on both indicators. These results indicate that both *τ* and ChlF contribute to the *V*_cmax_ estimations.

Potential ill‐posedness could be reduced by introducing prior information on *V*_cmax_; for example, based on vegetation indices or pigment concentrations, of which Chl concentration is a most valid candidate (Houborg *et al*., 2015; Gitelson *et al*., 2016). A more mechanistic modelling could potentially reduce the uncertainty in other parameters, such as $K_{n}^{0}$ and *ς*. For example, the description of energy partitioning to NPQ used in this study could be replaced with MD12, whereas models developed by Zaks *et al*. (2013) and Matuszyńska *et al*. (2016) may help constrain *ς* using fluorescence kinetics.

The residuals of two datasets (chamber vs leaf clip) used with method 3 (Figs 5i, 9) have the same span, which is encouraging, as it shows that similar results can be achieved with different types of spectral measurements (i.e. reflectance or transmittance). In general, predictions of *A* are at least 10% more accurate for estimations from light‐response curves compared with CO_2_ responses; and similarly, light‐response curves provide a higher accuracy of *V*_cmax_ retrieval.

Our study showed that a quantification of photosynthesis from transmittance or reflectance and ChlF during light‐ and CO_2_‐response curves is possible and very promising.

## Author contributions

NV is the main author of this paper; she collected the datasets, coupled the models, and performed the analysis. CvdT and WV assisted with model development, sensitivity analysis, and manuscript development.
